# A Novel Data Augmentation Method for Radiomics Analysis Using Image Perturbations

**DOI:** 10.1007/s10278-024-01013-0

**Published:** 2024-05-06

**Authors:** F Lo Iacono, R. Maragna, G. Pontone, V. D. A. Corino

**Affiliations:** 1 Department of Electronics, Information and Bioengineering, Politecnico Di Milano, Milan, Italy; 2 Department of Perioperative Cardiology and Cardiovascular Imaging, Centro Cardiologico Monzino IRCCS, Milan, Italy; 3 Department of Biomedical, Surgical and Dental Sciences, University of Milan, Milan, Italy

**Keywords:** Radiomics, Data augmentation, Data balancing, ROI perturbation, Cardiac amyloidosis

## Abstract

Radiomics extracts hundreds of features from medical images to quantitively characterize a region of interest (ROI). When applying radiomics, imbalanced or small dataset issues are commonly addressed using under or over-sampling, the latter being applied directly to the extracted features. Aim of this study is to propose a novel balancing and data augmentation technique by applying perturbations (erosion, dilation, contour randomization) to the ROI in cardiac computed tomography images. From the perturbed ROIs, radiomic features are extracted, thus creating additional samples. This approach was tested addressing the clinical problem of distinguishing cardiac amyloidosis (CA) from aortic stenosis (AS) and hypertrophic cardiomyopathy (HCM). Twenty-one CA, thirty-two AS and twenty-one HCM patients were included in the study. From each original and perturbed ROI, 107 radiomic features were extracted. The CA-AS dataset was balanced using the perturbation-based method along with random over-sampling, adaptive synthetic (ADASYN) and the synthetic minority oversampling technique (SMOTE). The same methods were tested to perform data augmentation dealing with CA and HCM. Features were submitted to robustness, redundancy, and relevance analysis testing five feature selection methods (*p*-value, least absolute shrinkage and selection operator (LASSO), semi-supervised LASSO, principal component analysis (PCA), semi-supervised PCA). Support vector machine performed the classification tasks, and its performance were evaluated by means of a 10-fold cross-validation. The perturbation-based approach provided the best performances in terms of f1 score and balanced accuracy in both CA-AS (f1 score: 80%, AUC: 0.91) and CA-HCM (f1 score: 86%, AUC: 0.92) classifications. These results suggest that ROI perturbations represent a powerful approach to address both data balancing and augmentation issues.

**Supplementary Information:**

The online version contains supplementary material available at 10.1007/s10278-024-01013-0.

## Introduction

Radiomics is an emerging field of research focused on extracting quantitative data from medical images with the aim to deliver an even more accurate personalized medicine. Radiomics models have been widely employed in oncology, due to their utility in classification of tumors [1, 2], prediction of treatment response [3–5], and prognostication [6], but, recently, it has gained increasing interest for the diagnosis and assessment of prognosis of cardiovascular disease [7–14].

Dealing with medical images datasets, common challenges are usually represented by class unbalance or lack of data: the reasons of these issues lie in the low prevalence of some diseases, poor medical equipment or resources, difficulties in obtaining images that meet specific, or issues related to the complex and time-consuming tasks of image labeling and segmentation [15]. Imbalanced or small dataset may negatively affect machine learning model performance leading to overfitting, biased outcomes, and inaccurate results. Therefore, it is crucial to address these issues when training machine learning models.

As concern imbalanced datasets, resampling procedures are usually applied as pre-processing step: these methods can be organized into under-sampling, oversampling and hybrid methods [16].

Under-sampling achieves class balancing by removing some instances from the majority class. Several under-sampling methods, with different filtering principles, have been implemented: random Under-Sampling, Near Miss, Tomek Links, and Edited Nearest Neighbors (ENN) [17, 18]. Random Under-Sampling discards random samples from the majority class, Near Miss selects majority samples close to some minority samples, Tomek Links defines two instances as boundary or noisy instances used to remove samples from the majority class, while ENN tests each instance, using k Nearest Neighbors (kNN), with the rest of the samples, and discards them if incorrectly classified [16].

Employing over-sampling methods, indeed, balancing is reached by generating new data based on samples from the minority class. Within this resampling class, Synthetic Minority Over-Sampling Technique (SMOTE) is the most employed method which employs a kNN approach to produce new minority instances between the existing ones. Other over-sampling techniques have been tested, as random over-sampling [16], adaptive synthetic (ADASYN), and borderline SMOTE [17, 18]. Random over-sampling (ROS) copies random minority class samples while ADASYN is based on SMOTE and creates new instances according to the data distribution and density, thus generating different numbers of new samples. Borderline SMOTE performs SMOTE on borderline samples, which are instances that are often misclassified by their nearest neighbors [16].

Finally, hybrid systems involve a combination of minority class over-sampling with majority class under-sampling. These methods include SMOTE-ENN, which combines SMOTE for over-sampling and ENN for under-sampling, and SMOTE-Tomek, which uses SMOTE for over-sampling and Tomek Links for under-sampling. These two hybrid methods were employed in [17] and [18] with the purpose to balance the training dataset and remove the noisy points at the wrong side of the decision boundary, finding better models with good generalization ability [16].

Dealing with small dataset, data augmentation strategies are generally used on the training data to reduce the risk of overfitting. Data augmentation is commonly used in the field of deep learning, as it requires a lot of training samples to produce an effective model. Augmentation is usually achieved in two ways: by generating different images from the original images, such as cropped images with different locations, noise-added images, or mirrored images, or using generative adversarial network (GAN) [19]. To the best of our knowledge, no studies, dealing with medical imaging, has performed features augmentation in the machine learning field. In [20] only, the authors proposed a new data augmentation technique employing the Conditional Wasserstein GAN. This approach consists of two networks, a generator and a discriminator, which work together to generate realistic-like EEG features. However, it should be noted that all the resampling methods previously cited might be employed to perform data augmentation.

In this study, both class imbalance and small dataset issues were addressed, dealing with radiomic-based model aimed to differentiating cardiac amyloidosis (CA) from aortic stenosis (AS) and hypertrophic cardiomyopathy (HCM). As demonstrated in [21] and [22], in fact, radiomics represents a promising non-invasive tool to conduct a differential diagnosis of CA using medical images, such as cardiac computed tomography (CCT), acquired in the clinical routine.

CA is an infiltrative disease characterized by the extracellular deposition of misfolded amyloidogenic proteins. It is an underestimated cause of heart failure and cardiac arrhythmias often only diagnosed at autopsy post-mortem [23]. Due to its phenotypical features, as the increased biventricular wall thickness, myocardial stiffening, and restrictive physiology of the left and right ventricles, CA has often been misdiagnosed as AS, or HCM [24, 25]. AS is the most common valvular heart disease, triggered by a progressive aortic valve narrowing, leading to heart failure, while HCM is a common inherited cardiovascular disease whose main macroscopic characteristics are myocardial wall thickening and stiffening, and myocyte hypertrophy. Due to the absence of specific symptoms, and the need of specific diagnostic tools, not available in some cases, the diagnosis of CA is difficult and time-consuming. In addition, the differentiation of this relatively rare disease from other pathologies is extremely important for the diverse therapeutic options and the difference in long-term prognosis [26, 27]. Lastly, the recent emergence of novel and effective treatments, improving the prognosis of patients with CA, has significantly enhanced the importance of early detection of this disease.

The aim of this work it to develop new balancing and augmentation methods, starting from region of interest (ROI) perturbations. In particular, ROIs were perturbed, i.e., slightly modified by applying geometrical operations, namely, erosion, dilation and contour randomization, and the features extracted from the perturbed ROIs are used to balance or augment the original data. Thus, the final aim of the study is to investigate the impact of this novel image-based method and compare it with the most commonly used data-based resampling methods, i.e., ROS, ADASYN, and SMOTE, in two classification tasks: distinguishing CA from AS and CA from HCM. In the first case, perturbations are employed to balance the dataset while in the former they are used to perform data augmentation.

In addition, the developed method was validated by performing data augmentation on an external set, including cardiac magnetic resonance (CMR) images for analyzing epicardial adipose tissue (EAT). EAT is the fat depot existing between the heart muscle’s surface and the inner layer of the pericardium and its biological activity can potentially influence major health issues like coronary artery disease, atrial fibrillation, and heart failure. In the context of EAT, literature reports promising results in classifying and predicting atrial fibrillation using radiomic features [28–30]. In the current study, we addressed a different classification task involving the identification of patients experiencing a hard cardiac event (HCE).

## Materials and Methods

### CCT Acquisition Protocol

CCT examinations were performed using using 256-slices (Revolution CT; GE Healthcare, Milwaukee, WI) or 320-slices wide volume coverage CT scanner (Aquilion ONE VisionTM; Canon Medical Systems Corp., Tokyo, Japan).

No premedication with beta-blockers or nitrates was added before CT acquisition. Patients received a fixed dose of 50 ml bolus of contrast medium (400 mg of iodine per milliliter, Iomeprol; Bracco, Milan, Italy) despite the BMI via antecubital vein at an infusion rate of 5 ml s^−1^ followed by 50 ml of saline solution at 5 ml·s^−1^.

### CMR Acquisition Protocol

CMR studies were performed with a 1.5-T Discovery MR450 (G.E. Healthcare, Milwaukee, Wisconsin). Breath-hold steady-state free-precession cine imaging was performed in vertical and horizontal long- and short-axis orientations.

A contrast-enhanced breath-hold segmented T1-weighted inversion-recovery gradient-echo sequence was used for the detection of late gadolinium enhancement. Steady state-free precession cine sequences were acquired using the following parameters: echo time 1.57 ms, 15 segments, repetition time 46 ms without view sharing, slice thickness 8 mm, field of view 350 × 263 mm, and pixel size 1.4 × 2.2 mm. Late gadolinium enhancement imaging was performed 10 to 20 min after administration of an intravenous bolus of gadolinium at a flow rate of 4 mL/s followed by saline flush. The inversion time was individually adjusted to null normal myocardium (usual range 220 to 300 ms). The following parameters were used: FOV: 380 to 420 mm; TR/TE: 4.6/1.3 ms; *α*: 20°; matrix: 256 × 192; S.T.: 8 mm; and no interslice gap.

### Study Population and Baseline Characteristics

The study included 21 patients affected by CA, 21 patients with HCM and 32 patients with AS. In all patients, CA was detected through bone scintigraphy using 3,3-diphosphono-1,2-propanodicarboxylicacid (DPD) and confirmed with myocardial biopsy (Congo red and immunohistochemical staining) in case of uncertain diagnosis. In AS patients, the concomitant presence of CA was excluded by bone scintigraphy and/or cardiac magnetic resonance (CMR). All patients underwent comprehensive evaluation with transthoracic echocardiography using commercially available equipment (iE33 or Epiq, Philips Medical System, or Vivid-9, GE Healthcare) measuring LV end-diastolic (LVEDV) and end-systolic (LVESV) volumes indexed for body surface area, LV ejection fraction (LVEF), and intraventricular septum thickness (IVS). Clinical characteristics are shown in Table 1.

**Table 1:** Baseline characteristics of study population (cardiac computed tomography images)

	**AS**	**HCM**	**CA**
Number of patients	32	21	21
Age, years	82 (78–84)	62 (53–73)	74 (67–76)**, §
Male	21 (65%)	12 (57%)	7 (33%)
Body surface area, m^2^	1.8 (1.7–1.9)	1.85 (1.78–2)	1.9 (1.7–2)
Body mass index, kg/m^2^	26 (25–29)	25 (23–28)	26 (22–32)
LVESVi, ml/m^2^	22 (18–26)	15 (24–23)	18 (15–24)
LVEDVi, ml/m^2^	58 (46–66)	50 (40–55)	36 (29–54)**, §
LVEF, %	62(57–65)	64 (59–68)	48 (43–58)**, §§
IVS thickness, mm	13 (12–14)	18 (12–19)	16 (12–17)*

Values are expressed as absolute number and percentage or median and interquartile range

*CA* cardiac amyloidosis, *AS* aortic stenosis, *HCM* hypertrophic cardiomyopathy, *LVESVi* left ventricle end-diastolic volume index for body surface area, *LVEDVi* left ventricle end-systolic volume index for body surface area, *LVEF* left ventricle ejection fraction, *IVS* intraventricular septum thickness

*p < 0.05 CA vs. AS, **p < 0.01 CA vs. AS, § *p* < 0.05 CA vs. HCM, §§ *p* < 0.01 CA vs. HCM

The external validation set included 40 patients, 20 of them experienced an HCE. These patients were referred to perform clinically indicated dipyridamole stress CMR for suspected or known coronary artery disease between January 2011 and December 2014 at Centro Cardiologico Monzino (Milan, Italy). Clinical characteristics are shown in Table 2.

**Table 2:** Baseline characteristics of study population (cardiac magnetic resonance images)

	**HCE**	**No HCE**
Number of patients	20	20
Age, years	63 (57–70)	65 (59–73)
Male	16 (80%)	16 (80%)
Body mass index, kg/m^2^	24 (23–27)	27 (25–27)
CMR left ventricular ejection fraction (%)	60 (54–70)	64 (56–68)
Familiar history (y/n)	2 (10%)	2 (10%)
Smoking (y/n)	7 (80%)	6 (80%)
Hypertension (y/n)	15 (75%)	10 (50%)
Hyperlipemia (y/n)	13 (65%)	12 (60%)
Diabetes (y/n)	3 (15%)	3 (15%)

*HCE* hard cardiac event, *CMR* cardiac magnetic resonance

The institutional Ethical Committee approved the studies, and all the patients signed the informed consent.

### Image Processing and Radiomics Feature Extraction

CCT images were manually segmented by an expert cardiac imager with level III European Association of Cardiovascular Imaging to obtain the left ventricular wall. Two image preprocessing steps were performed: a 3D Gaussian filter with a 3 × 3 × 3 voxel kernel and *σ* = 0.5 was applied to denoise the images, and a B-spline interpolation was performed to resample voxel size to an isotropic resolution of 2 mm (as in [30]).

EAT was manually segmented by an expert cardiac imager with level III European Association of Cardiovascular. Image pre-processing was performed to denoise images using a 3D Gaussian filter with a 3 × 3 × 3 voxel kernel and σ = 0.5. Intensity-non uniformities were corrected using the N4ITK algorithm and intensity standardization was performed with z-score. Finally, B-spline interpolation was employed to resample the voxel size to a 2-mm isotropic resolution.

MATLAB R2017a (MathWorks, Natick, MA, USA) was used to perform all the previous steps.

From each dataset, a total of 107 radiomic features were extracted from the segmented ROIs using Pyradiomics 3.0 [31]. The features obtained belong to three categories: shape and size (14 features), first order statistics (18 features), and texture (75 features), being the latter based on the grey level co-occurrence matrix (GLCM), grey level run length matrix (GLRLM), and gray level size zone matrix (GLSZM).

### Radiomic Feature Evaluation

Radiomic features underwent a series of feature selection steps based on robustness, redundancy, and relevance analysis.

**Fig. 1:**
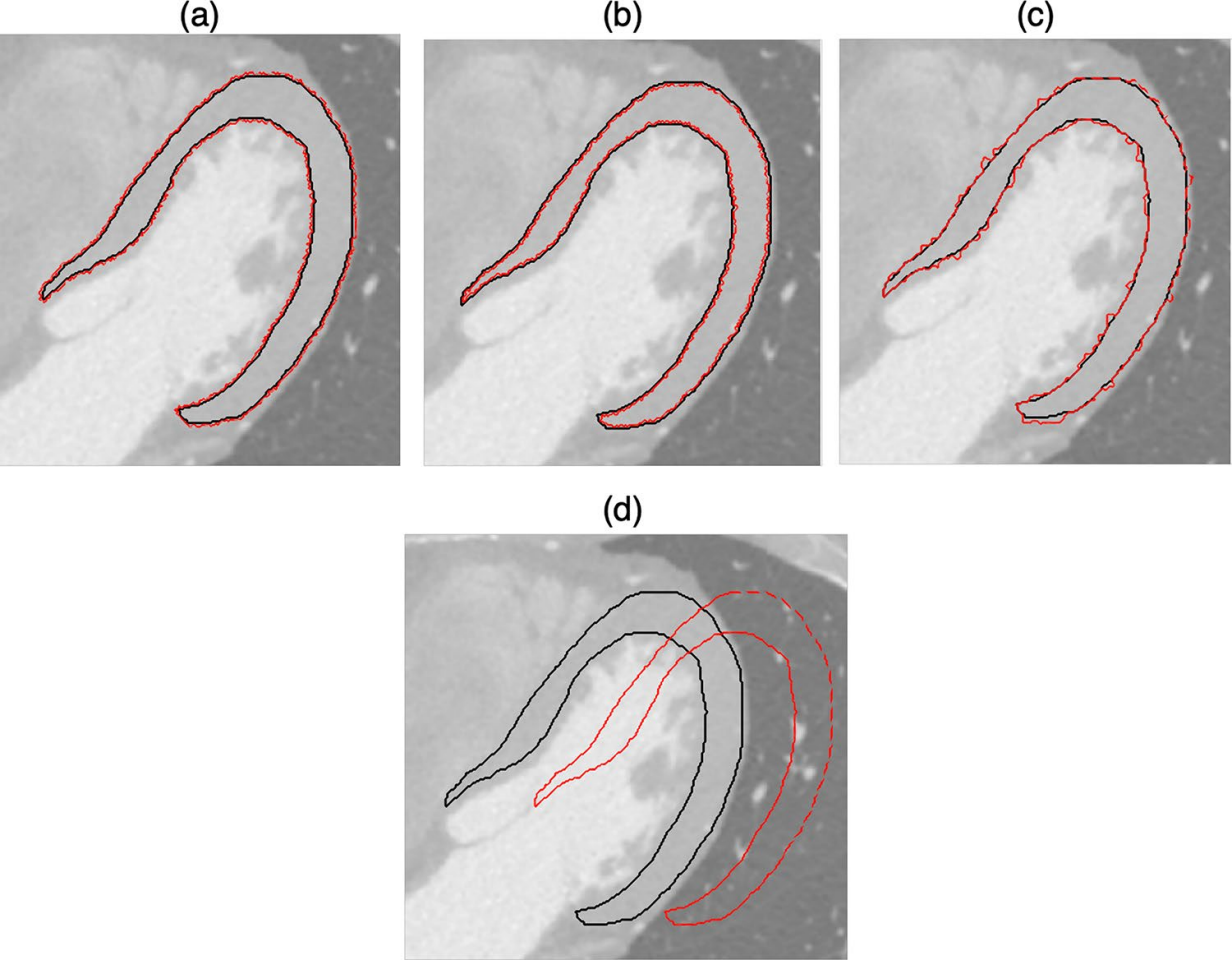
Example of perturbations applied to the ROI. The filled structure represents the original ROI, while the dashed red line represents the modified ROI used to assess features stability. **a)** ROI dilation (zoom-in with τ  = 0.15). **b)** ROI erosion (zoom-out with τ =  − 0.15). **c**) ROI contour randomization with superpixels area equal to 25 mm^2^. **d**) ROI translation of 30% in the x positive direction

To evaluate the robustness, i.e., stability and discriminative capability of extracted features, ROI small and large perturbations were performed following the approaches described in [32] and [33]. The underlying hypothesis is that small perturbations mimic the effects of random multiple delineations while large transformations help to identify non-discriminant features. In particular, three small entity perturbations were used to assess feature stability, namely erosion, dilation, and contour randomization, while a large translation was performed to assess feature discrimination capacity (see Fig. 1). Only first-order and textural features were submitted to this evaluation.

The entire workflow is implemented in MATLAB 2022a (Mathworks, Natick, MA, USA) and applied to each single CCT slice.

**Erosion** and **dilation** were used to reduce and enlarge the segmentation mask, respectively. These transformations are achieved by employing a kernel, represented as a matrix of 0 s and 1 s, which slides across the image. For this study, a 3 × 3 kernel was created, encompassing a pixel p with coordinates (x, y), and its two horizontal neighbors [(*x* + 1, *y*), (*x* − 1, *y*)] and two vertical neighbors [(*x*, *y* + 1), (*x*, *y* − 1)]. During the erosion process, a pixel in the original image is set to 1 only if all the pixels covered by the kernel are also 1. On the other hand, in ROI dilation, a pixel in the original image (either 1 or 0) is set to 1 if at least one of the pixels covered by the kernel is 1. The final area achieved is A_f_ = [A_0_ (1 + τ)], being V_0_ the original volume and τ > 0 the growth factor while τ < 0 the shrinkage factor. In this study, the parameter |τ| is set to 0.15, resulting in a ROI that is reduced or enlarged by 15% of the original ROI area.

**Contour randomization** is achieved using a superpixel-based segmentation strategy, where superpixels represent connected clusters of pixels with similar intensity characteristics (in this study, the area occupied by a superpixel was set to 25 mm^2^). The process of generating a randomized contour involves comparing these superpixels to the original segmentation mask. To accomplish this, the degree of overlap ν between each superpixel and the original ROI contour is considered. Superpixels with an overlap ν ≥ 0.90 are included in the modified ROI, while those with an overlap ν ≤ 0.20 are excluded. For superpixels with an overlap value between 0.20 and 0.90, a random selection process is applied to determine their inclusion in the new ROI.

Finally, to evaluate feature discrimination capacity, the ROIs were subjected to **translation**, which involves shifting them along both the x and y axes. A large translation was applied, corresponding to ± 30% of the length of the bounding box surrounding each ROI. Four translations were computed for each ROI: two in the positive direction and two in the negative direction, separately for both the x and y directions.

Features extracted from the original and the modified ROI were compared using the intraclass correlation coefficient (ICC). The ICC was computed following the approach described in [33]. According to general guidelines [34], robustness assessment was performed using two thresholds: features having an ICC > 0.75 for small entity perturbation (erosion, dilation, and random contour) were considered stable, while features with an ICC < 0.5, for large entity translations, were considered discriminative.

Features selected as stable, and discriminant were thus submitted to redundancy analysis. The Spearman correlation coefficient *ρ* was calculated for each couple of features. If |*ρ*| exceeded a predefined threshold, only features with the lower mean Spearman coefficient when compared to all other n-2 (*n* = total number of features) were kept. Following the results achieved in [22], two thresholds were considered: 0.90 and 0.95.

Finally, the most relevant features were selected by testing 5 different feature selection methods: *p*-value, least absolute shrinkage and selection operator (LASSO), semi-supervised LASSO (ssLASSO), principal component analysis (PCA), semi-supervised PCA (ssPCA). The *p*-value and semi-supervised based methods involves conducting a Mann–Whitney U test on each feature to identify significant differences between two unpaired groups of patients and then to apply the LASSO or PCA.

### ML Model Development

In this study, two classification tasks were considered: distinguishing CA from AS and CA from HCM.

For both the classification tasks, the first step performed in the model development process was dividing the dataset into training and test set by mean of a 10-fold cross validation. In each fold, training set was used to select the final set of features and train the classifier, while the test set was employed to evaluate model performance. Since [22] reported support vector machine (SVM) as the best classifier in distinguishing CA from AS, it was chosen to address the two tasks.

During the training phase, data were balanced or augmented using features extracted from the perturbated ROI. In particular, dilated, eroded, and randomized ROI were employed. ROI dilation and erosion were performed using |τ|= 0.30, thus, obtaining a ROI shrunk or enlarged by 30%. ROI contour randomization was, indeed, performed using a superpixel area of 4 cm^2^ which provided a 70% of overlapping between the original and the modified ROI. Compared to robustness analysis, larger perturbation entities were chosen to generate new and different instances contributing to introduce variability and prevent overfitting.

### Data Balancing

**Fig. 2:**
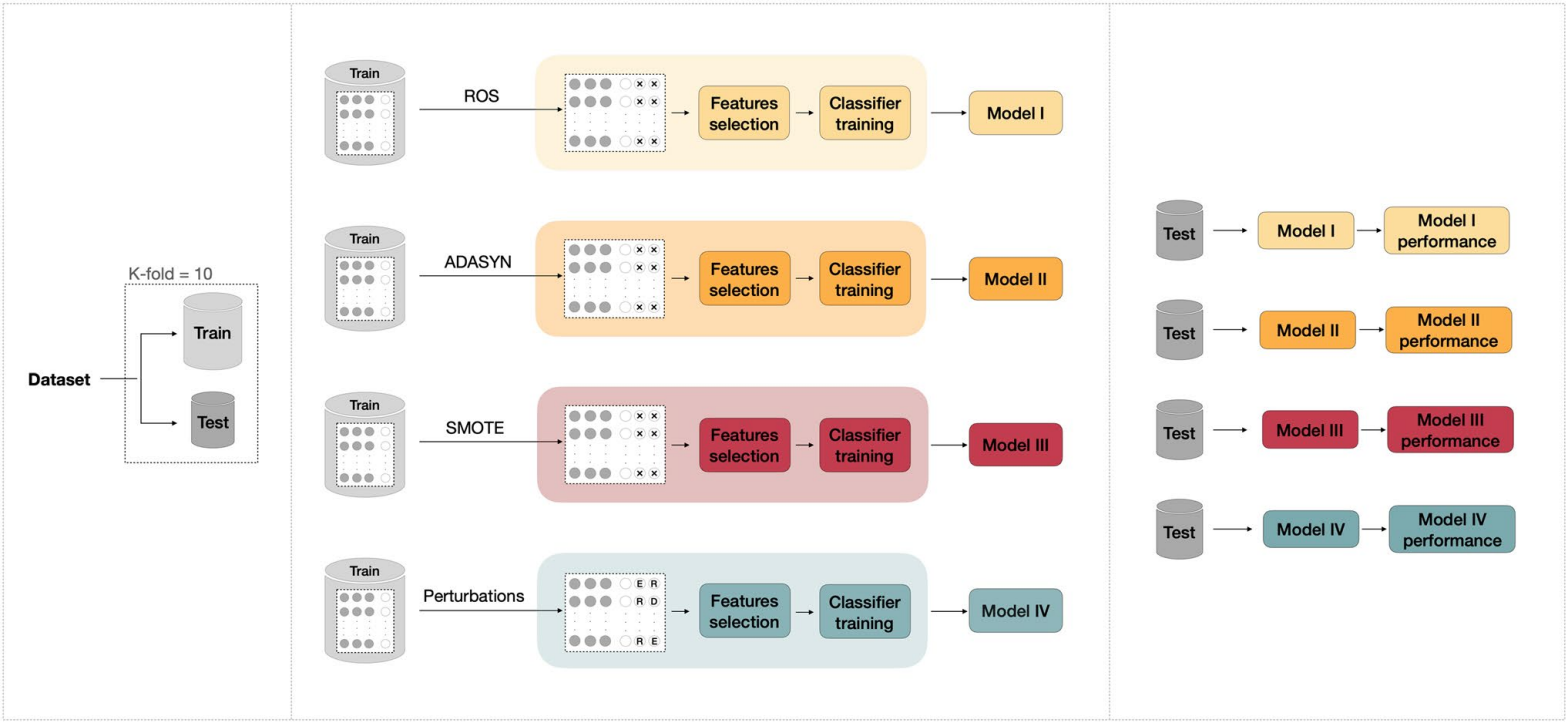
Machine learning workflow dealing with data-balancing task. In the training set, the grey dots represent majority class samples, whereas the white ones represent minority class samples. X: a synthetic sample obtained by using ROS, ADASYN, or SMOTE, E: a synthetic sample obtained from an eroded ROI, D: a synthetic sample obtained from a dilated ROI, R: a synthetic sample obtained from a random contour perturbation of the ROI. ROS: random over-sampling; ADASYN: adaptive synthetic; SMOTE: synthetic minority oversampling technique

In the CA-AS classification task, since the dataset was imbalanced (32 AS vs. 21 CA), balancing was performed by two methods, summarized in Fig. 2, both applied to the training set. In the first approach, ROS, ADASYN, and SMOTE, commonly used as data balancing techniques, were employed. ROS randomly selects samples from the minority class, with replacement, and adds them to the training dataset. ADASYN uses a weighted distribution for minority class examples based on their learning difficulty and produces more synthetic data for challenging instances and less for easier-to-learn minority samples. SMOTE creates synthetic samples for the minority class by interpolating between existing instances. The algorithm selects the k-nearest neighbors for each minority instance and generates new synthetic instances along the line segments connecting the instance and its neighbors. In this work, k was set to 5.

The second approach, indeed, employed ROI perturbations. The balancing required number of patients was selected from the minority class in the training set. Thus, ROIs belonging to such patients were perturbated randomly choosing among erosion, dilation, and contour randomization. Finally, features were extracted from the perturbed ROIs and added to the training set.

### Data Augmentation

**Fig. 3:**
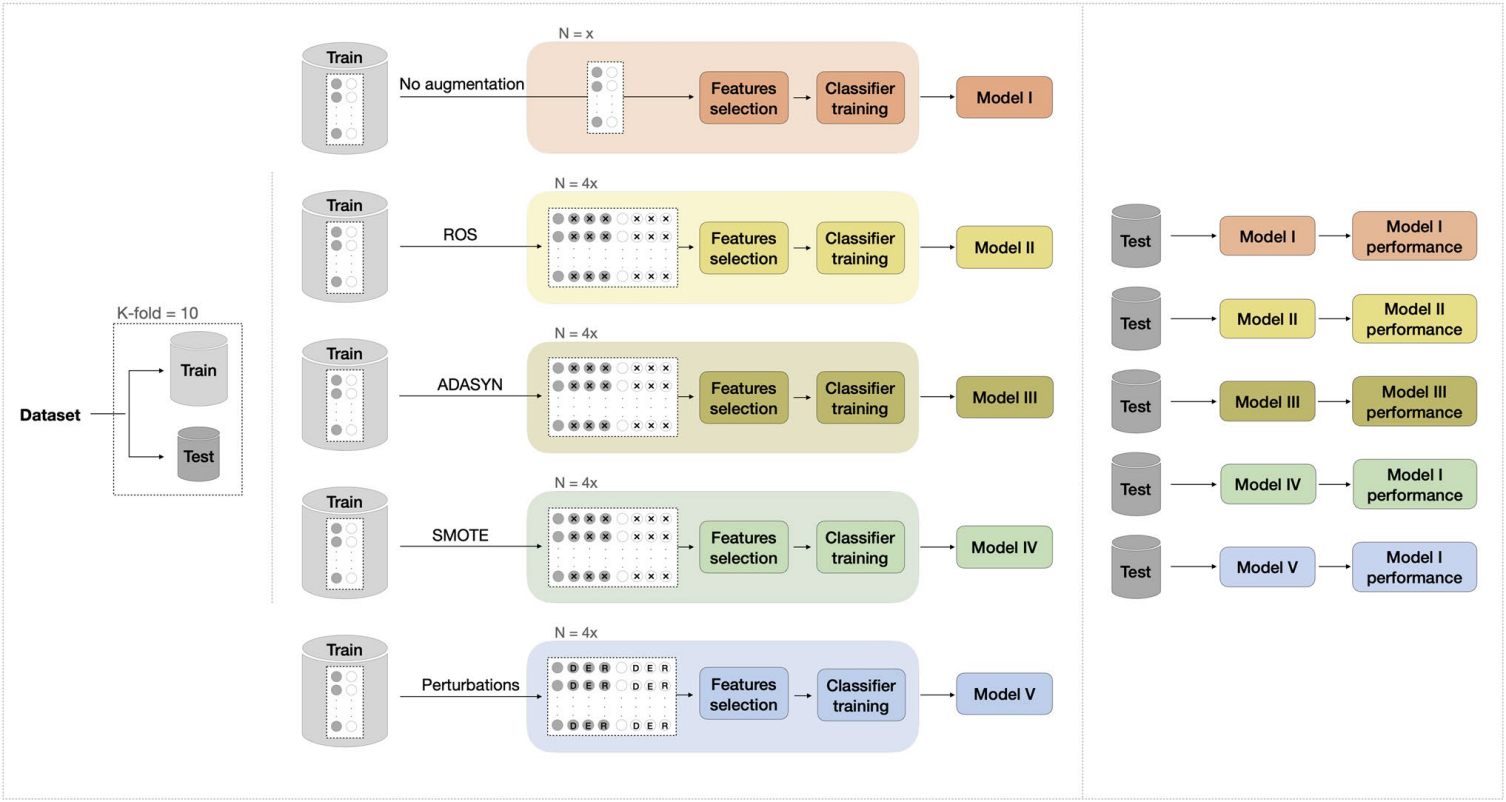
Machine learning workflow dealing with data augmentation task. In the training set, the grey and dots represent the two classes to be classified. X: a synthetic sample obtained by using ROS, ADASYN, or SMOTE, E: a synthetic sample obtained from an eroded ROI, D: a synthetic sample obtained from a dilated ROI, R: a synthetic sample obtained from a random contour perturbation of the ROI. ROS: random over-sampling; ADASYN: adaptive synthetic; SMOTE: synthetic minority oversampling technique

As concern CA-HCM classification, since the dataset was balanced but small, data augmentation was performed using ROS, ADASYN, SMOTE, or perturbations, and results were compared to the non-augmented data. The three methods are schematized in Fig. 3.

Employing ROS, ADASYN, and SMOTE method, the samples belonging to a single class were quadrupled, and the algorithm was applied: the process was carried out independently for both classes, and the newly generated samples were then integrated into the training set.

In the perturbation approach, indeed, augmentation was performed by adding the features extracted from the perturbed ROIs pertained to the training samples: in such a way, for each training instance, three new samples obtained from the dilated, eroded, and randomized ROI were obtained. Thus, the samples belonging to each class were quadrupled as when using the other balancing techniques.

### Validation Model

To validate the proposed data augmentation method, an external set of EAT CMR images was considered to classify patients experiencing an HCE from patients who did not. Features extracted from the original ROIs were submitted to the three feature selection steps described in the radiomic feature evaluation section, to obtain a set of robust, non-redundant, and relevant features. The correlation threshold was set to 0.95.

As stated for the CA-AS and CA-HCM datasets, a  10-fold cross validation was used to divide the dataset into training and test set: within each fold the training set was employed to select the final features set and train the SVM model, while the test set to evaluate the classifier performance. As the dataset was balanced but small, data augmentation was performed according to the workflow explained in the data augmentation section.

## Results

### Feature Selection

A total of 32 and 40 features were identified as robust, i.e., stable, and discriminative, for CA-AS and CA-HCM classification tasks, respectively (Table 3).

**Table 3:** Number of features selected by the different feature selection methods as a function of the used correlation threshold and the data balancing method (mean ± standard deviation)

Task: CA vs. AS				
Feature selection method	**Correlation threshold = 0.95**			
	ROS	ADASYN	SMOTE	Perturbations
*p*-value	10 ± 2	9 ± 2	9 ± 2	9 ± 3
LASSO	7 ± 4	6 ± 3	7 ± 3	10 ± 2
ssLASSO	7 ± 2	5 ± 1	6 ± 2	5 ± 1
PCA	7 ± 0	7 ± 0	7 ± 0	8 ± 0
ssPCA	5 ± 0	5 ± 1	4 ± 1	5 ± 1

*CA* cardiac amyloidosis, *AS* aortic stenosis, *ROS* random over-sampling, *ADASYN* adaptive synthetic, *SMOTE* synthetic minority oversampling technique, *LASSO* least absolute shrinkage and selection operator, *ssLASSO* semi-supervised LASSO, *PCA* principal component analysis, *ssPCA*, semi-supervised PCA

Performing the distinction between CA and AS, using a correlation threshold of 0.95, an average of 23 ± 1, 23 ± 1, 23 ± 1, and 24 ± 1 non-redundant features were selected at each train-test split, for the ROS, ADASYN, SMOTE, and the perturbation-based balancing methods respectively.

Dealing with CA-HCM classification, the redundancy analysis with correlation threshold of 0.95 identified 28 ± 1, 28 ± 1, 27 ± 1, 26 ± 1, and 30 ± 0 non-correlated features using the no-augmentation, ROS, ADASYN, SMOTE, and perturbation-based augmentation methods, respectively.

Focusing on the five feature selection methods, the average number of features identified as relevant from each technique, are reported in Table 4 and 5. As shown, in the case of CA–AS differentiation, the number of selected features ranges between 5 and 10, in the CA-HCM issue, between 6 and 23.

**Table 4:** Number of features selected by the different feature selection methods, using a correlation threshold of 0.95, as a function of the data augmentation method (mean ± standard deviation)

Task: CA vs. HCM					
Feature selection method	**Correlation threshold = 0.95**				
	No augmentation	ROS	ADASYN	SMOTE	Perturbations
*p*-value	13 ± 2	21 ± 2	20 ± 2	21 ± 1	23 ± 1
LASSO	9 ± 3	13 ± 1	13 ± 1	13 ± 1	13 ± 1
ssLASSO	8 ± 2	11 ± 2	11 ± 2	11 ± 1	11 ± 1
PCA	8 ± 0	8 ± 0	8 ± 0	8 ± 1	9 ± 0
ssPCA	6 ± 0	7 ± 1	7 ± 0	6 ± 0	8 ± 0

*CA* cardiac amyloidosis, *HCM* hypertrophic cardiomyopathy, *ROS* random over-sampling, *ADASYN* adaptive synthetic, *SMOTE* synthetic minority oversampling technique, *LASSO* least absolute shrinkage and selection operator, *ssLASSO* semi-supervised LASSO, *PCA* principal component analysis, *ssPCA*, semi-supervised PCA

**Table 5:** Number of features selected by the different feature selection methods, using a correlation threshold of 0.95, as a function of the data augmentation method (mean ± standard deviation) in the validation set

Task: HCE vs. not-HCE					
Feature selection method	**Correlation threshold = 0.95**				
	No augmentation	ROS	ADASYN	SMOTE	Perturbations
*p*-value	12 ± 2	30 ± 4	28 ± 3	34 ± 4	24 ± 2
LASSO	6 ± 4	18 ± 1	19 ± 2	19 ± 1	22 ± 2
ssLASSO	5 ± 1	11 ± 2	15 ± 2	14 ± 2	13 ± 3
PCA	12 ± 0	11 ± 0	11 ± 0	11 ± 1	14 ± 0
ssPCA	6 ± 1	10 ± 0	9 ± 1	9 ± 1	10 ± 1

*HCE* hard cardiac event, *ROS* random over-sampling, *ADASYN* adaptive synthetic, *SMOTE* synthetic minority oversampling technique, *LASSO* least absolute shrinkage and selection operator, *ssLASSO* semi-supervised LASSO, *PCA* principal component analysis, *ssPCA* semi-supervised PCA

Results for the correlation threshold equal to 0.90 are shown in the Supplementary material.

### Classification with Data Balancing

**Fig. 4:**
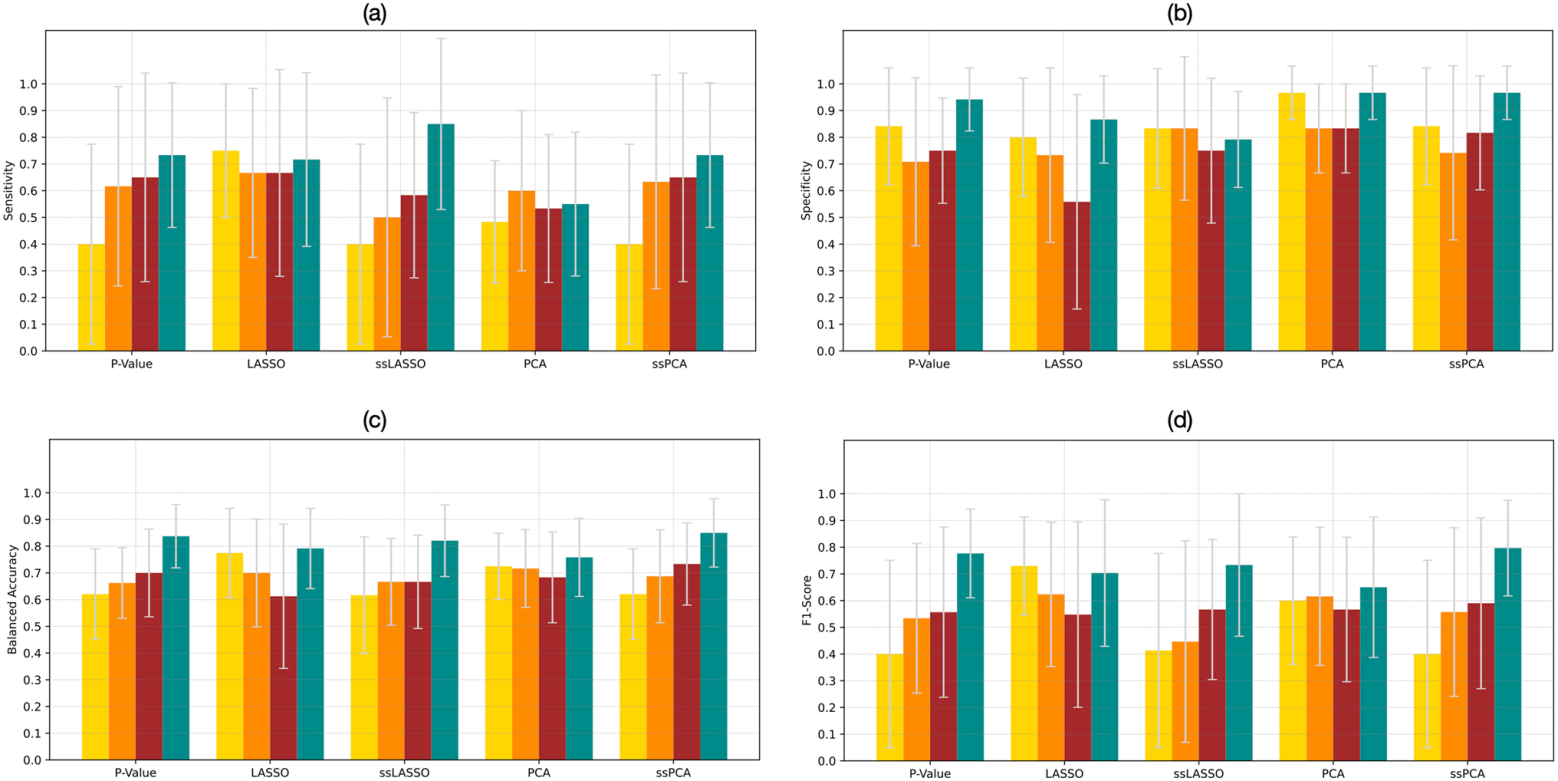
Mean **a) **sensitivity, **b) **specificity, **c) **balanced accuracy, and **d) **f1 score, averaged on the 10 test folds for the different feature selection methods when differentiating CA from AS, using ROS (yellow bars), ADASYN (orange bars), SMOTE (red bars), or ROI perturbations (green bars). LASSO, least absolute shrinkage and selection operator; ssLASSO, semi-supervised LASSO; PCA, principal component analysis; ssPCA, semi-supervised PCA. ROS: random over-sampling; ADASYN: adaptive synthetic; SMOTE: synthetic minority oversampling technique; LASSO: least absolute shrinkage and selection operator; ssLASSO: semi-supervised LASSO; PCA: principal component analysis; ssPCA: semi-supervised PCA

The performance achieved for the classification task CA vs. AS, by combining SVM with the 0.95 correlation threshold and each of the five feature selection methods is reported in Fig. 4 in terms of sensitivity, specificity, balanced accuracy, and f1 score, averaged on the 10 test folds. The analogous figures for the correlation threshold 0.90 are shown in Supplementary Fig. 1. It can be noted that the highest mean f1 score and balanced accuracy, 80% and 85%, respectively, were reached using the ssPCA feature selection technique and ROI perturbations balancing method. These results were compared with the ones achieved by the other balancing methods using a repeated measure ANOVA, or Kruskal Wallis test, followed by post hoc tests. Balanced accuracy was significantly higher using the perturbation-based method than all the other balancing techniques, f1 score was significantly higher using the perturbation-based method than using ROS and SMOTE. Considering all the feature selection methods and all the performance metrics, the differences were not always statistically significant, but, in most cases, the performance metrics of the perturbation-based balancing method tend to be superior to those with ROS, ADASYN, and SMOTE.

**Fig. 5:**
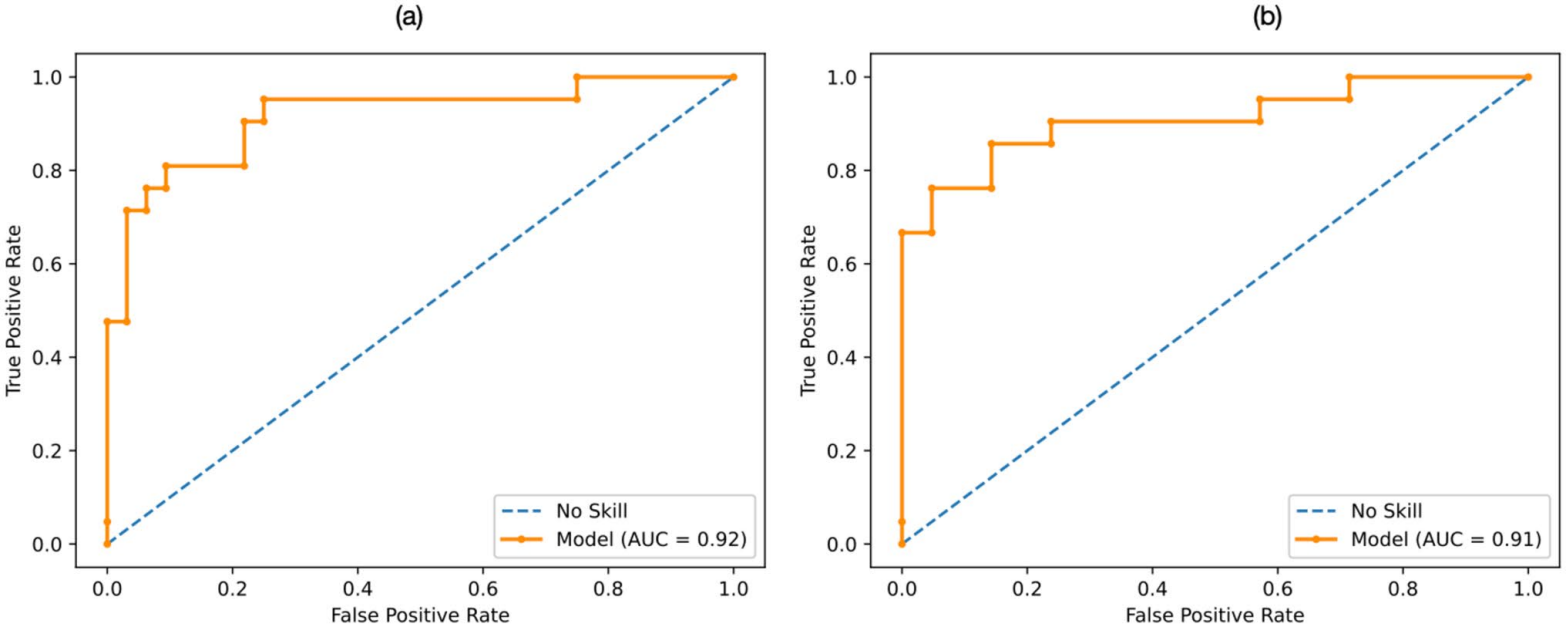
ROC curve differentiating **a) **CA from AS computed using the ssPCA method as feature selection and **b) **CA from HCM using the PCA method as feature selection, in both cases using ROI perturbations

Figure 5(a) shows the ROC curve, highlighting an AUC of 0.92.

### Classification with Data Augmentation

**Fig. 6:**
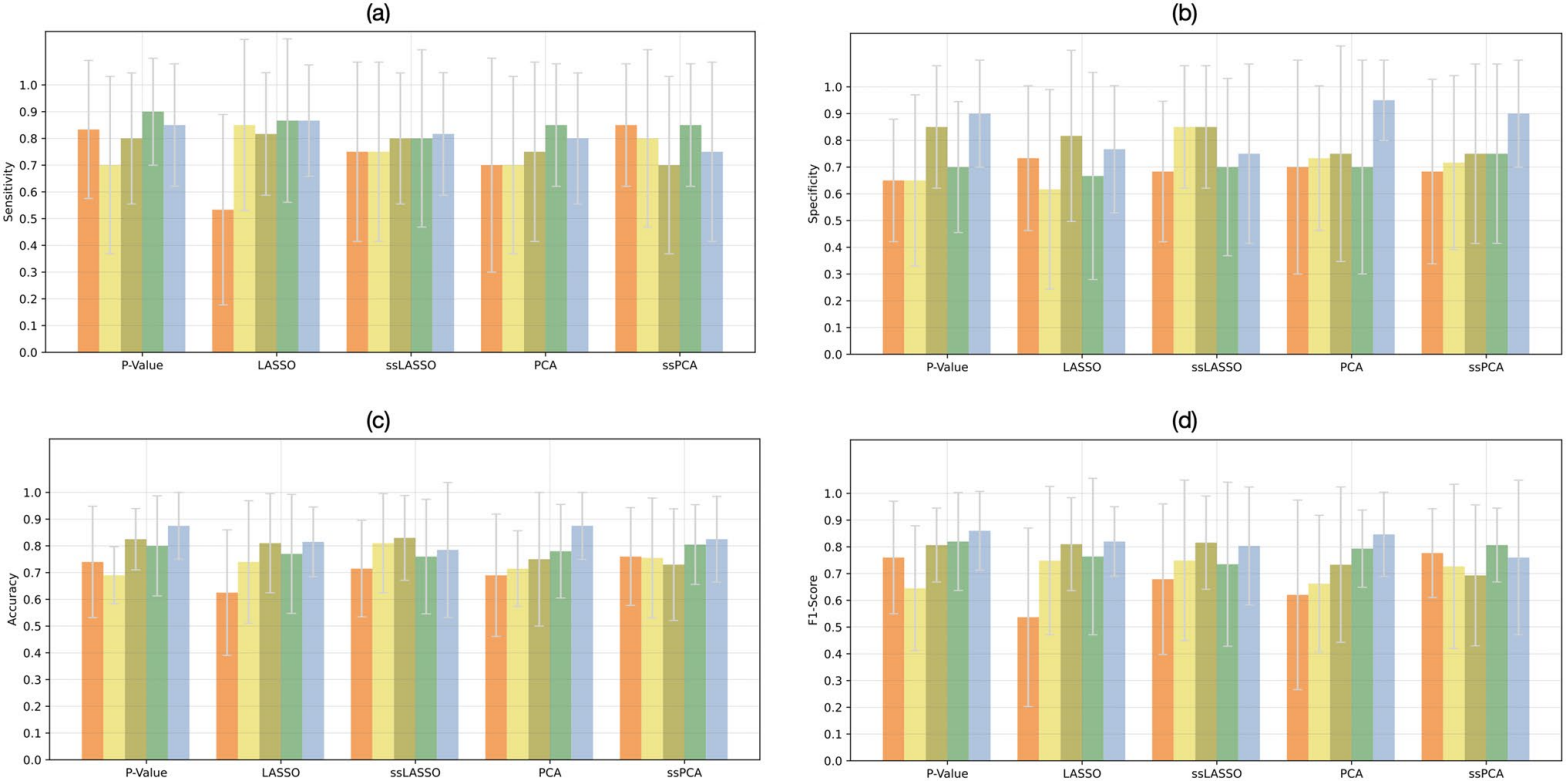
Mean **a) **sensitivity, **b)** specificity, **c)** balanced accuracy, **d) **f1 score, averaged on the 10 test folds for the different feature selection methods when differentiating CA from HCM, without augmentation (orange bars), using ROS (light yellow bars), ADASYN (light green bars), and SMOTE (dark green bars) or ROI perturbations (light blue bars). ROS: random over-sampling; ADASYN: adaptive synthetic; SMOTE: synthetic minority oversampling technique; LASSO, least absolute shrinkage and selection operator; ssLASSO, semi-supervised LASSO; PCA, principal component analysis; ssPCA, semi-supervised PCA

The performance achieved for the classification task CA vs. HCM, by combining SVM with the 0.95 correlation threshold and each of the five feature selection methods, is reported in Fig. 6 in terms of sensitivity, specificity, accuracy, and f1 score, averaged on the 10 test folds. The analogous figures for the correlation threshold 0.90 are shown in in Supplementary Fig. 2. It can be noted that all performance metrics tend to be higher when using data augmentation in respect to the original dataset. Moreover, in most cases, the perturbation-based method tends to achieve higher f1 score and accuracy compared to ROS, ADASYN, and SMOTE (no significant differences). In particular, the highest mean f1 score and accuracy, 86% and 88%, respectively, were reached using the *p*-value feature selection technique and perturbation-based augmentation method. Figure 5(b) shows the ROC curve, highlighting an AUC of 0.91.

### Validation Set

A total of 100 features were identified as robust, i.e., stable, and discriminative. At each train-test split an average of 62 ± 1, 62 ± 2, 62 ± 1, 60 ± 2, and 61 ± 1 non-redundant features, using the no-augmentation, ROS, ADASYN, SMOTE, and perturbation-based augmentation methods, respectively.

Focusing on the five feature selection methods, the average number of features, identified as relevant from each technique, is reported in Table 5.

**Fig. 7:**
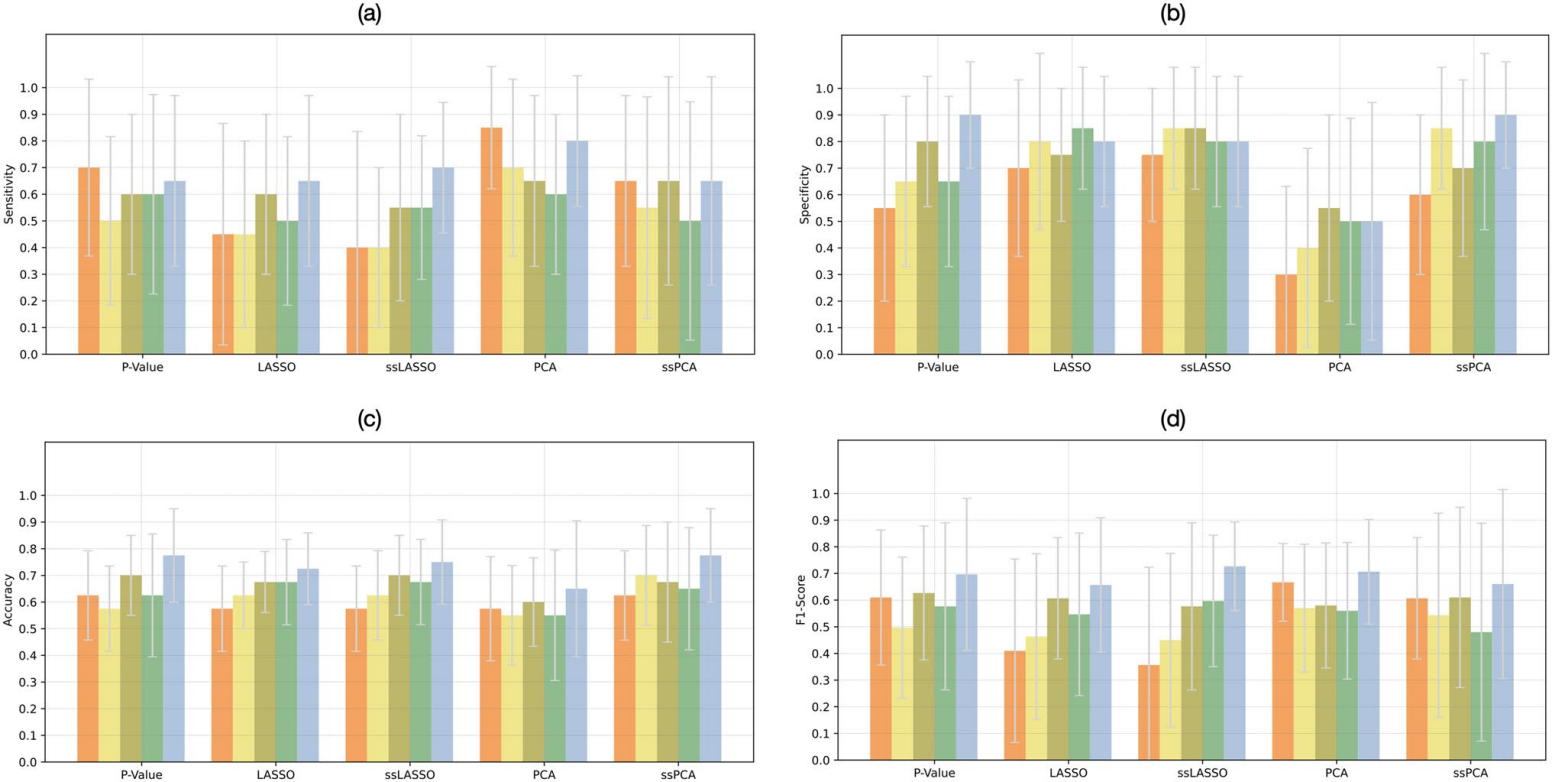
Mean **a) **sensitivity, **b) **specificity, **c) **balanced accuracy, and **d) **f1 score, averaged on the 10 test folds for the different feature selection methods when differentiating patients experiencing an HCE from patients who did not, without augmentation (orange bars), using ROS (light yellow bars), ADASYN (light green bars) and SMOTE (dark green bars) or ROI perturbations (light blue bars). ROS: random over-sampling; ADASYN: adaptive synthetic; SMOTE: synthetic minority oversampling technique; LASSO, least absolute shrinkage and selection operator; ssLASSO, semi-supervised LASSO; PCA, principal component analysis; ssPCA, semi-supervised PCA

The performance achieved for the classification task HCE vs. not-HCE, by combining SVM with the 0.95 correlation threshold and each of the five feature selection methods, is reported in Fig. 7 in terms of sensitivity, specificity, accuracy, and f1 score, averaged on the 10 test folds. Comparing the different augmentation techniques, it can be observed that in most cases f1 score and balanced accuracy show higher values using the perturbation-based method (no significant differences), with maximum scores, 78% and 70%, respectively, reached in correspondence of the *p*-value feature selection method.

## Discussions

The main findings of this study are the following: (i) in case of imbalanced dataset, ROI perturbations can be used to balance the two classes achieving greater performance compared to ROS, ADASYN, and SMOTE; (ii) in case of small dataset, ROI perturbations can be used to increase data size providing better results compared to a no-augmentation or a ROS, ADASYN, or SMOTE-based approach.

In clinical settings, many medical image datasets present challenges such as imbalance or lack of data, which can make it difficult to achieve high predictive performance using data-driven machine learning methods. Since radiomics focus on medical images, these are common issues found within several studies [17, 18, 35].

Dealing with imbalanced dataset, three resampling approaches are usually chosen as a pre-processing step: over-sampling, under-sampling, and hybrid systems. The over-sampling technique helps to improve the performance of models but present the drawback of overfitting and introduces additional noise. Under-sampling method, indeed, works by removing instances from the majority class showing an advantage in saving computation time [18]. However, it might remove critical instances required for defining the class boundaries and might leave unnecessary instances of the majority class in the training data, generating additional noise [36]. Hybrid systems, finally, combined the two previously described resampling techniques.

In the radiomic field, literature reports several studies applying various resampling methods. In [17], Xie et al. investigated the impact of ten re-sampling techniques on PET-based radiomics models for predicting prognosis in head and neck cancer. The overall survival and disease-free survival were predicted in three imbalanced datasets finding that resampling techniques impacted positively on the prediction performance, but depending on the clinical problem and dataset, the results of each individual balancing approach can vary. The study of Park et al. [35] involved the application of ROS and SMOTE resampling techniques in radiomics analysis for predicting the grade and histological subtype of meningiomas. SMOTE resampling demonstrated higher performance, with an AUC of 0.86, with respect to ROS (AUC = 0.85). In [18] the authors have focused on predicting lymph node metastasis in clinical stage T1 lung adenocarcinoma patients using radiomics analysis based on PET/CT images: the unbalanced dataset problem in this study was addressed by testing ten resampling strategies (ROS, ADASYN, SMOTE, bSMOTE, RUS, NM, TL, ENN, SMOTE-TL, SMOTE-ENN) among which ENN resampling method showed prediction performances averagely higher of 0.04 ± 0.02 (AUC = 0.71). It can be observed that there is no consensus concerning the best resampling method as this depends on the clinical problem and the data intrinsic characteristics, such as, dataset size and dimensionality, imbalance ratio, overlapping between classes or borderline samples [18].

Working with small dataset, indeed, data augmentation strategies are useful to increase the training data and mitigate the potential for overfitting. However, data augmentation is mainly used in the deep learning field, and it is usually achieved by generating new transformed versions of the original images or using GAN. Literature lacks medical images-based studies reporting methods addressing feature augmentation, but this task can be also achieved by employing several resampling methods.

In the current study, a new image perturbation-based technique was developed to address, for the first time, the class balancing and data augmentation issues. Differently from other existing methods, this approach focuses on generating new features starting from the image itself and not from the available features. Two correlation thresholds (0.90 and 0.95) and five feature selection methods (*p*-value, LASSO, ssLASSO, PCA, ssPCA) were combined and tested to evaluate the results among different models. Finally, to assess the efficacy of the proposed perturbation-based method, its performances were compared with those obtained by using SMOTE. The performance metrics of both classifications (CA vs. HCM and CA vs. AS) show that using a correlation threshold equal to 0.95, in most cases, our perturbation-based method outperforms SMOTE in terms of both f1 score and balanced accuracy, independently from the chosen feature selection approach. The same observation can be done setting the correlation threshold to 0.90 (Supplementary material).

In particular, dealing with CA-AS classification, the perturbation-based balancing strategy reached the highest balanced accuracy value of 85%, in correspondence of the ssPCA, while the maximum accuracy achieved by ROS, ADASYN, and SMOTE was 78% (LASSO model), 72% (PCA model), 73% (ssPCA model), respectively. As concern the CA-HCM classification the greatest accuracy of 88% was obtained with the perturbation-based method using the *p*-value and the PCA feature selection methods while ROS and ADASYN achieved their best accuracy values, 80% and 81%, in correspondence of ssLASSO and the no-augmentation and the SMOTE-based approaches showed the highest accuracy of 76% and 81%, respectively, using the ssPCA. The promising results reached by the developed data augmentation method, observed in both the CA-AS and CA-HCM classification tasks were finally validated with an external CMR dataset. Distinguishing patients experiencing an HCE from those who did not, the perturbation-based method demonstrated higher performances than all the other methods. In particular, perturbation-based performance metrics reached the maximum accuracy value of 78%, in correspondence of the p-value and ssPCA-based feature selection methods, while no augmentation, ROS, ADASYN, and SMOTE reached a maximum accuracy of 63%, 70%, 70%, and 68%, respectively.

CA patients were included in both classification tasks, as identifying this disease represents a relevant and challenging task from the clinical point of view. Nowadays, an accurate diagnosis of CA is challenging and crucial as this disease is substantially underdiagnosed and mimics a wide range of cardiac and systemic conditions. Furthermore, the recent introduction of new and effective therapies has emphasized the increased importance of its early recognition to improve patient prognosis. Endomyocardial biopsy is the historic gold standard for diagnosis of CA, but it carries a small risk of serious complication and requires the technical expertise of a procedural cardiologist. Over the last decade, the great progress achieved in the cardiac imaging field has opened new frontiers in the diagnosis of CA. Bone scintigraphy has emerged as a reliable non-invasive alternative to endomyocardial biopsy [37], but the approach presents several limitations from the logistic and economic point of view.

Literature shows that several studies have tried to improve CA diagnosis using machine learning algorithms. In [38], Wu et al. developed a machine learning tool able to distinguish 74 patients with CA from 64 patients with HCM with an AUC of 0.86, using speckle tracking echocardiography data. The same clinical goal was achieved in [39] where the authors combined CMR-based radiomic texture features with conventional MR metrics reaching an accuracy of 0.85 in classifying 85 patients affected by CA and 82 affected by HCM. Radiomics has recently achieved good results in differentiating CA from AS. In [22], a CCT-based radiomic model was employed to distinguish 15 patients affected by CA from 15 patients with AS with an accuracy of 0.83. Similar results were reached in [21] with an AUC of 0.92 considering 21 patients with CA and 44 with AS. These results are in line with the ones obtained in the present study.

## Conclusions

ROI perturbations can be used to balance an imbalanced dataset as well as to augment data size in a radiomics-based classification study.

## Supplementary Information

Below is the link to the electronic supplementary material.

Supplementary file1 (DOCX 410 KB)

## Data Availability

The raw data supporting the conclusions of this article will be made available by the authors, upon reasonable request.
