# Supplementary material for: A Novel Data Augmentation Method for Radiomics Analysis Using Image Perturbations
Source: J Imaging Inform Med. 2024 May 6;37(5):2401–14. doi: 10.1007/s10278-024-01013-0 (PMC11522260; doi:10.1007/s10278-024-01013-0)

Supplementary material

**Results for correlation threshold equal to 0.90**

Performing the distinction between CA and AS, using a correlation threshold of 0.90, an average of 23 ± 1, 23 ± 1, 23 ± 1 and 24 ± 1 non-redundant features were selected at each train-test split, for the ROS, ADASYN, SMOTE and the perturbations-based balancing methods respectively.

Dealing with CA-HCM classification, the redundancy analysis with correlation threshold of 0.90 identified 28 ± 1, 28 ± 1, 27 ± 1, 26 ± 1 and 30 ± 0 non-correlated features using the no-augmentation, ROS, ADASYN, SMOTE and perturbations-based augmentation methods, respectively.

**Table 1**: Number of features selected by the different feature selection methods using a correlation threshold of 0.90, as a function of used the data augmentation method (mean ± standard deviation).

| Task | Features selection method | **Correlation threshold = 0.90** | | | |
| --- | --- | --- | --- | --- | --- |
|  |  | ROS | ADASYN | SMOTE | Perturbations |
| CA vs AS | p-value | 9 ± 2 | 8 ± 1 | 8 ± 1 | 9 ± 2 |
|  | LASSO | 7 ± 3 | 6 ± 3 | 7 ± 2 | 10 ± 3 |
|  | ssLASSO | 6 ± 1 | 5 ± 2 | 6 ± 1 | 5 ± 1 |
|  | PCA | 7 ± 0 | 7 ± 0 | 7 ± 0 | 8 ± 0 |
|  | ssPCA | 5 ± 1 | 5 ± 1 | 5 ± 1 | 5 ± 1 |

**Table 2**: Number of features selected by the different feature selection methods using a correlation threshold of 0.90, as a function of used the data augmentation method (mean ± standard deviation).

| Task | Features selection method | **Correlation threshold = 0.90** | | | | |
| --- | --- | --- | --- | --- | --- | --- |
|  |  | No augmentation | ROS | ADASYN | SMOTE | Perturbations |
| CA vs HCM | p-value | 11 ± 1 | 18 ± 2 | 19 ± 2 | 17 ± 1 | 23 ± 1 |
|  | LASSO | 10 ± 3 | 12 ± 0 | 12 ± 1 | 10 ± 1 | 12 ± 1 |
|  | ssLASSO | 7 ± 1 | 10 ± 1 | 10 ± 2 | 10 ± 1 | 11 ± 1 |
|  | PCA | 8 ± 0 | 8 ± 1 | 8 ± 0 | 7 ± 1 | 9 ± 0 |
|  | ssPCA | 6 ± 0 | 7 ± 1 | 7 ± 0 | 6 ± 1 | 8 ± 0 |

**Table 3**: Sensitivity, specificity, balanced accuracy and f1 score, averaged on the 10 test folds for different feature selection methods and a correlation threshold of 0.90, when differentiating CA from AS, using ROS, ADASYN, SMOTE or ROI perturbations (mean ± standard deviation).

| **Task: CA vs AS** | | | | | |
| --- | --- | --- | --- | --- | --- |
| Features selection method | Metrics | **Correlation threshold = 0.90** | | | |
|  |  | ROS | ADASYN | SMOTE | Perturbations |
| p-value | Sensitivity | 45 ± 42 % | 61 ± 37 % | 65 ± 32 % | 73 ± 27 % |
|  | Specificity | 78 ± 21 % | 78 ± 30 % | 75 ± 20 % | 97 ± 10 % |
|  | Balanced accuracy | 61 ± 25 % | 72 ± 14 % | 70 ± 15 % | 85 ± 13 % |
|  | F1-score | 40 ± 36 % | 67 ± 14 % | 59 ± 25 % | 80 ± 18 % |
| LASSO | Sensitivity | 62 ± 37 % | 62 ± 37 % | 57 ± 42 % | 67 ± 39 % |
|  | Specificity | 70 ± 35 % | 80 ± 22 % | 74 ± 29 % | 90 ± 15 % |
|  | Balanced accuracy | 66 ± 26 % | 71 ± 18 % | 65 ± 23 % | 78 ± 19 % |
|  | F1-score | 57 ± 33 % | 58 ± 33 % | 53 ± 36 % | 66 ± 36 % |
| ssLASSO | Sensitivity | 55 ± 42 % | 55 ± 42 % | 65 ± 39 % | 85 ± 32 % |
|  | Specificity | 80 ± 22 % | 80 ± 27 % | 77 ± 21 % | 79 ± 18 % |
|  | Balanced accuracy | 68 ± 16 % | 68 ± 18 % | 71 ± 18 % | 82 ± 16 % |
|  | F1-score | 49 ± 35 % | 51 ± 35 % | 59 ± 32 % | 74 ± 29 % |
| PCA | Sensitivity | 52 ± 23 % | 55 ± 35 % | 53 ± 28 % | 50 ± 32 % |
|  | Specificity | 97 ± 10 % | 83 ± 17 % | 83 ± 17 % | 90 ± 21 % |
|  | Balanced accuracy | 74 ± 13 % | 69 ± 16 % | 68 ± 17 % | 70 ± 17 % |
|  | F1-score | 63 ± 24 % | 55 ± 32 % | 57 ± 27 % | 55 ± 31 % |
| ssPCA | Sensitivity | 45 ± 35 % | 53 ± 36 % | 65 ± 32 % | 73 ± 27 % |
|  | Specificity | 80 ± 22 % | 78 ± 30 % | 78 ± 26 % | 97 ± 10 % |
|  | Balanced accuracy | 63 ± 14 % | 65 ± 17 % | 71 ± 14 % | 85 ± 13 % |
|  | F1-score | 43 ± 32 % | 51 ± 30 % | 61 ± 25 % | 80 ± 18 % |

CA: cardiac amyloidosis; HCM: hypertrophic cardiomyopathy; ROS: random over-sampling; ADASYN: adaptive synthetic; SMOTE: synthetic minority oversampling technique; LASSO: least absolute shrinkage and selection operator; ssLASSO: semi-supervised LASSO; PCA: principal component analysis; ssPCA: semi-supervised PCA.

**Table 4**: Sensitivity, specificity, balanced accuracy and f1 score, averaged on the 10 test folds for different feature selection methods and a correlation threshold of 0.95, when differentiating CA from AS, using ROS, ADASYN, SMOTE or ROI perturbations (mean ± standard deviation).

| **Task: CA vs AS** | | | | | |
| --- | --- | --- | --- | --- | --- |
| Features selection method | Metrics | **Correlation threshold = 0.95** | | | |
|  |  | ROS | ADASYN | SMOTE | Perturbations |
| p-value | Sensitivity | 40 ± 37 % | 61 ± 37 % | 65 ± 39 % | 73 ± 27 % |
|  | Specificity | 84 ± 22 % | 71 ± 31 % | 75 ± 20 % | 94 ± 12 % |
|  | Balanced accuracy | 62 ± 17 % | 66 ± 13 % | 70 ± 16 % | 83 ± 12 % |
|  | F1-score | 40 ± 35 % | 53 ± 28 % | 56 ± 32 % | 78 ± 17 % |
| LASSO | Sensitivity | 75 ± 25 % | 67 ± 32 % | 67 ± 39 % | 72 ± 33 % |
|  | Specificity | 80 ± 22 % | 73 ± 33 % | 55 ± 40 % | 87 ± 16 % |
|  | Balanced accuracy | 78 ± 17 % | 70 ± 20 % | 61 ± 27 % | 79 ± 15 % |
|  | F1-score | 73 ± 18 % | 62 ± 27 % | 55 ± 35 % | 70 ± 27 % |
| ssLASSO | Sensitivity | 40 ± 37 % | 50 ± 45 % | 58 ± 31 % | 85 ± 32 % |
|  | Specificity | 83 ± 22 % | 83 ± 27 % | 75 ± 27 % | 79 ± 18 % |
|  | Balanced accuracy | 62 ± 22 % | 67 ± 16 % | 67 ± 17 % | 82 ± 13 % |
|  | F1-score | 41 ± 36 % | 45 ± 38 % | 57 ± 26 % | 73 ± 27 % |
| PCA | Sensitivity | 48 ± 23 % | 60 ± 30 % | 53 ± 28 % | 55 ± 27 % |
|  | Specificity | 97 ± 10 % | 83 ± 17 % | 83 ± 17 % | 97 ± 10 % |
|  | Balanced accuracy | 72 ± 12 % | 73 ± 15 % | 68 ± 17 % | 76 ± 15 % |
|  | F1-score | 60 ± 24 % | 62 ± 26 % | 57 ± 27 % | 75 ± 26 % |
| ssPCA | Sensitivity | 40 ± 37 % | 63 ± 40 % | 65 ± 39 % | 73 ± 27 % |
|  | Specificity | 84 ± 22 % | 74 ± 33 % | 82 ± 21 % | 97 ± 10 % |
|  | Balanced accuracy | 69 ± 17 % | 62 ± 17 % | 73 ± 15 % | 85 ± 13 % |
|  | F1-score | 40 ± 35 % | 56 ± 32 % | 59 ± 32 % | 80 ± 18 % |

CA: cardiac amyloidosis; HCM: hypertrophic cardiomyopathy; ROS: random over-sampling; ADASYN: adaptive synthetic; SMOTE: synthetic minority oversampling technique; LASSO: least absolute shrinkage and selection operator; ssLASSO: semi-supervised LASSO; PCA: principal component analysis; ssPCA: semi-supervised PCA.

**Table 5**: Sensitivity, specificity, balanced accuracy and f1 score, averaged on the 10 test folds for different feature selection methods and a correlation threshold of 0.90, when differentiating CA from HCM, without augmentation, using ROS, ADASYN, SMOTE or ROI perturbations (mean ± standard deviation).

| **Task: CA vs HCM** | | | | | | |
| --- | --- | --- | --- | --- | --- | --- |
| Features selection method | Metrics | **Correlation threshold = 0. 90** | | | | |
|  |  | No Augmentation | ROS | ADASYN | SMOTE | Perturbations |
| p-value | Sensitivity | 83 ± 26 % | 70 ± 33 % | 70 ± 33 % | 90 ± 20 % | 85 ± 23 % |
|  | Specificity | 57 ± 35 % | 70 ± 33 % | 85 ± 23 % | 70 ± 24 % | 90 ± 20 % |
|  | Accuracy | 69 ± 25 % | 72 ± 8 % | 78 ± 18 % | 80 ± 19 % | 88 ± 13 % |
|  | F1-score | 76 ± 21 % | 65 ± 23 % | 81 ± 14 % | 82 ± 18 % | 88 ± 17 % |
| LASSO | Sensitivity | 58 ± 44 % | 75 ± 34 % | 77 ± 33 % | 87 ± 31 % | 87 ± 21 % |
|  | Specificity | 73 ± 27 % | 72 ± 33 % | 80 ± 33 % | 72 ± 33 % | 77 ± 24 % |
|  | Accuracy | 66 ± 26 % | 74 ± 17 % | 78 ± 17 % | 77 ± 22 % | 79 ± 20 % |
|  | F1-score | 54 ± 33 % | 75 ± 28 % | 81 ± 17 % | 76 ± 29 % | 81 ± 13 % |
| ssLASSO | Sensitivity | 75 ± 25 % | 75 ± 25 % | 85 ± 23 % | 75 ± 34 % | 82 ± 23 % |
|  | Specificity | 77 ± 24 % | 82 ± 32 % | 77 ± 23 % | 75 ± 25 % | 70 ± 33 % |
|  | Accuracy | 76 ± 16 % | 80 ± 18 % | 81 ± 22 % | 76 ± 32 % | 76 ± 19 % |
|  | F1-score | 68 ± 28 % | 85 ± 23 % | 82 ± 17 % | 74 ± 31 % | 76 ± 24 % |
| PCA | Sensitivity | 75 ± 34 % | 60 ± 38 % | 67 ± 39 % | 75 ± 34 % | 80 ± 24 % |
|  | Specificity | 55 ± 35 % | 78 ± 27 % | 75 ± 34 % | 65 ± 45 % | 87 ± 21 % |
|  | Accuracy | 65 ± 18 % | 69 ± 19 % | 71 ± 22 % | 78 ± 17 % | 71 ± 19 % |
|  | F1-score | 62 ± 35 % | 66 ± 26 % | 73 ± 29 % | 79 ± 14 % | 83 ± 11 % |
| ssPCA | Sensitivity | 90 ± 20 % | 60 ± 38 % | 65 ± 39 % | 80 ± 24 % | 80 ± 24 % |
|  | Specificity | 53 ± 28 % | 72 ± 33 % | 85 ± 23 % | 75 ± 34 % | 87 ± 21 % |
|  | Accuracy | 71 ± 18 % | 66 ± 28 % | 76 ± 22 % | 81 ± 15 % | 78 ± 13 % |
|  | F1-score | 78 ± 17 % | 73 ± 31 % | 69 ± 26 % | 81 ± 14 % | 83 ± 11 % |

CA: cardiac amyloidosis; HCM: hypertrophic cardiomyopathy; ROS: random over-sampling; ADASYN: adaptive synthetic; SMOTE: synthetic minority oversampling technique; LASSO: least absolute shrinkage and selection operator; ssLASSO: semi-supervised LASSO; PCA: principal component analysis; ssPCA: semi-supervised PCA.

**Table 6**: Sensitivity, specificity, balanced accuracy and f1 score, averaged on the 10 test folds for different feature selection methods and a correlation threshold of 0.95, when differentiating CA from HCM, without augmentation, using ROS, ADASYN, SMOTE or ROI perturbations (mean ± standard deviation).

| **Task: CA vs HCM** | | | | | | |
| --- | --- | --- | --- | --- | --- | --- |
| Features selection method | Metrics | **Correlation threshold = 0. 95** | | | | |
|  |  | No Augmentation | ROS | ADASYN | SMOTE | Perturbations |
| p-value | Sensitivity | 83 ± 26 % | 70 ± 33 % | 80 ± 24 % | 90 ± 20 % | 85 ± 23 % |
|  | Specificity | 65 ± 23 % | 65 ± 32 % | 85 ± 23 % | 70 ± 24 % | 90 ± 20 % |
|  | Accuracy | 74 ± 21 % | 69 ± 11 % | 83 ± 11 % | 80 ± 18 % | 88 ± 13 % |
|  | F1-score | 76 ± 21 % | 65 ± 23 % | 81 ± 14 % | 82 ± 18 % | 86 ± 15 % |
| LASSO | Sensitivity | 53 ± 36 % | 85 ± 32 % | 82 ± 23 % | 87 ± 31 % | 86 ± 21 % |
|  | Specificity | 73 ± 27 % | 62 ± 37 % | 82 ± 32 % | 67 ± 39 % | 77 ± 24 % |
|  | Accuracy | 63 ± 23 % | 74 ± 23 % | 81 ± 19 % | 77 ± 22 % | 82 ± 13 % |
|  | F1-score | 54 ± 33 % | 75 ± 28 % | 81 ± 17 % | 76 ± 29 % | 82 ± 13 % |
| ssLASSO | Sensitivity | 75 ± 34 % | 75 ± 34 % | 80 ± 24 % | 80 ± 33 % | 82 ± 23 % |
|  | Specificity | 68 ± 26 % | 85 ± 23 % | 85 ± 23 % | 70 ± 33 % | 75 ± 35 % |
|  | Accuracy | 72 ± 18 % | 81 ± 19 % | 83 ± 16 % | 76 ± 32 % | 79 ± 25 % |
|  | F1-score | 68 ± 28 % | 85 ± 23 % | 82 ± 17 % | 74 ± 31 % | 80 ± 22 % |
| PCA | Sensitivity | 70 ± 40 % | 70 ± 33 % | 75 ± 34 % | 85 ± 23 % | 80 ± 24 % |
|  | Specificity | 70 ± 40 % | 73 ± 27 % | 75 ± 40 % | 70 ± 40 % | 95 ± 15 % |
|  | Accuracy | 69 ± 23 % | 72 ± 14 % | 75 ± 25 % | 78 ± 17 % | 88 ± 13 % |
|  | F1-score | 62 ± 35 % | 66 ± 26 % | 73 ± 29 % | 79 ± 14 % | 86 ± 16 % |
| ssPCA | Sensitivity | 85 ± 23 % | 80 ± 33 % | 70 ± 33 % | 85 ± 23 % | 75 ± 34 % |
|  | Specificity | 68 ± 35 % | 72 ± 33 % | 75 ± 34 % | 75 ± 34 % | 90 ± 20 % |
|  | Accuracy | 76 ± 18 % | 76 ± 22 % | 73 ± 21 % | 81 ± 15 % | 83 ± 16 % |
|  | F1-score | 78 ± 17 % | 73 ± 31 % | 69 ± 26 % | 81 ± 14 % | 76 ± 29 % |

CA: cardiac amyloidosis; HCM: hypertrophic cardiomyopathy; ROS: random over-sampling; ADASYN: adaptive synthetic; SMOTE: synthetic minority oversampling technique; LASSO: least absolute shrinkage and selection operator; ssLASSO: semi-supervised LASSO; PCA: principal component analysis; ssPCA: semi-supervised PCA.

**Table 7**: Sensitivity, specificity, balanced accuracy and f1 score, averaged on the 10 test folds for different feature selection methods and a correlation threshold of 0.95, when differentiating HCE from not-HCE, without augmentation, using ROS, ADASYN, SMOTE or ROI perturbations (mean ± standard deviation).

| **Task: HCE vs not-HCE** | | | | | | |
| --- | --- | --- | --- | --- | --- | --- |
| Features selection method | Metrics | **Correlation threshold = 0.95** | | | | |
|  |  | No augmentation | ROS | ADASYN | SMOTE | Perturbations |
| p-value | Sensitivity | 70 ± 33 % | 50 ± 31 % | 60 ± 30 % | 60 ± 37 % | 65 ± 32 % |
|  | Specificity | 55 ± 35 % | 65 ± 32 % | 80 ± 24 % | 65 ± 32 % | 90 ± 20 % |
|  | Accuracy | 63 ± 17 % | 58 ± 16 % | 70 ± 15 % | 63 ± 23 % | 78 ± 18 % |
|  | F1-score | 61 ± 25 % | 50 ± 26 % | 63 ± 25 % | 58 ± 31 % | 70 ± 29 % |
| LASSO | Sensitivity | 45 ± 41 % | 45 ± 35 % | 60 ± 30 % | 50 ± 32 % | 65 ± 32 % |
|  | Specificity | 70 ± 33 % | 80 ± 33 % | 75 ± 25 % | 85 ± 23 % | 80 ± 24 % |
|  | Accuracy | 58 ± 16 % | 63 ± 12 % | 68 ± 11 % | 68 ± 16 % | 73 ± 13 % |
|  | F1-score | 41 ± 34 % | 46 ± 31 % | 61 ± 23 % | 55 ± 30 % | 66 ± 25 % |
| ssLASSO | Sensitivity | 40 ± 44 % | 40 ± 30 % | 55 ± 35 % | 55 ± 27 % | 70 ± 24 % |
|  | Specificity | 75 ± 25 % | 85 ± 23 % | 85 ± 23 % | 80 ± 24 % | 80 ± 24 % |
|  | Accuracy | 58 ± 16 % | 63 ± 17 % | 70 ± 15 % | 68 ± 16 % | 75 ± 16 % |
|  | F1-score | 36 ± 37 % | 45 ± 33 % | 58 ± 31 % | 60 ± 25 % | 73 ± 17 % |
| PCA | Sensitivity | 85 ± 23 % | 70 ± 33 % | 65 ± 32 % | 60 ± 30 % | 80 ± 24 % |
|  | Specificity | 30 ± 33 % | 40 ± 37 % | 55 ± 35 % | 50 ± 39 % | 50 ± 45 % |
|  | Accuracy | 58 ± 20 % | 55 ± 19 % | 60 ± 17 % | 55 ± 24 % | 65 ± 25 % |
|  | F1-score | 67 ± 15 % | 57 ± 24 % | 58 ± 23 % | 56 ± 26 % | 71 ± 20 % |
| ssPCA | Sensitivity | 65 ± 32 % | 55 ± 42 % | 65 ± 39 % | 50 ± 45 % | 65 ± 39 % |
|  | Specificity | 60 ± 30 % | 85 ± 23 % | 70 ± 33 % | 80 ± 33 % | 90 ± 20 % |
|  | Accuracy | 63 ± 17 % | 70 ± 19 % | 68 ± 23 % | 65 ± 23 % | 78 ± 18 % |
|  | F1-score | 61 ± 23 % | 54 ± 38 % | 61 ± 34 % | 48 ± 41 % | 66 ± 35 % |

HCE: hard cardiac event; ROS: random over-sampling; ADASYN: adaptive synthetic; SMOTE: synthetic minority oversampling technique; LASSO: least absolute shrinkage and selection operator; ssLASSO: semi-supervised LASSO, PCA: principal component analysis; ssPCA: semi-supervised PCA.

Fig.1: Mean (a) sensitivity, (b) specificity and (c) f1 score, averaged on the 10 test folds for different feature selection methods and a correlation threshold of 0.90, when differentiating CA from AS, using ROS (yellow bars), ADASYN (orange bars), SMOTE (red bars) or ROI perturbations (green bars). LASSO, least absolute shrinkage and selection operator; ssLASSO, semi-supervised LASSO; PCA, principal component analysis; ssPCA, semi-supervised PCA

(a) (b)

**
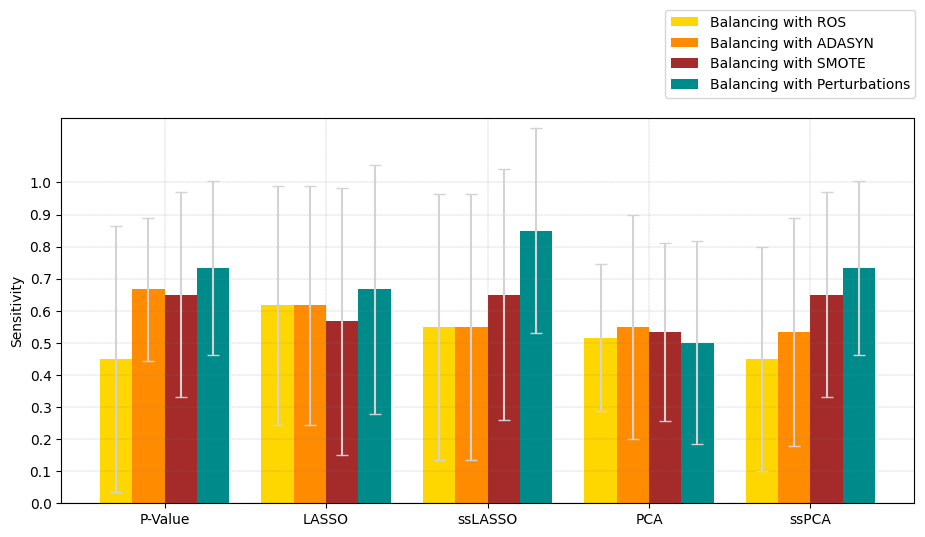

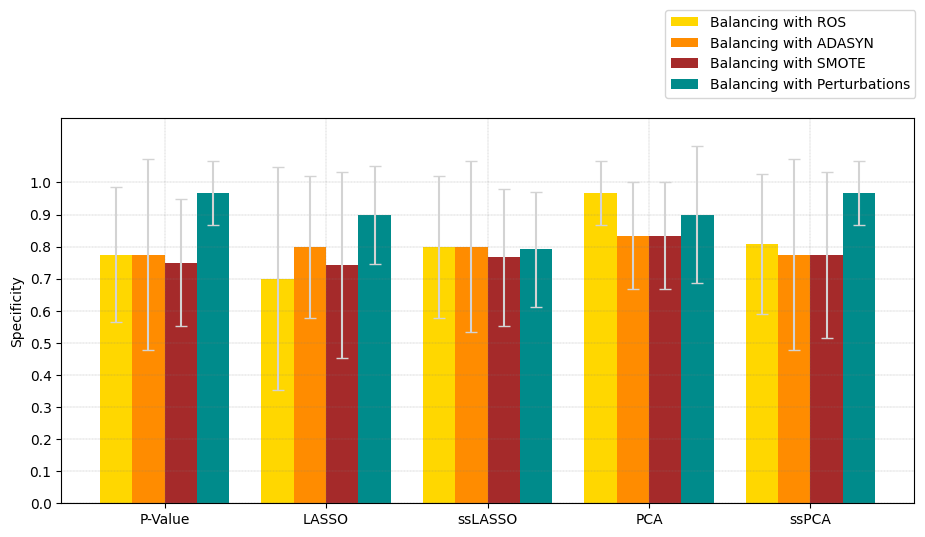
**

(c) (d)


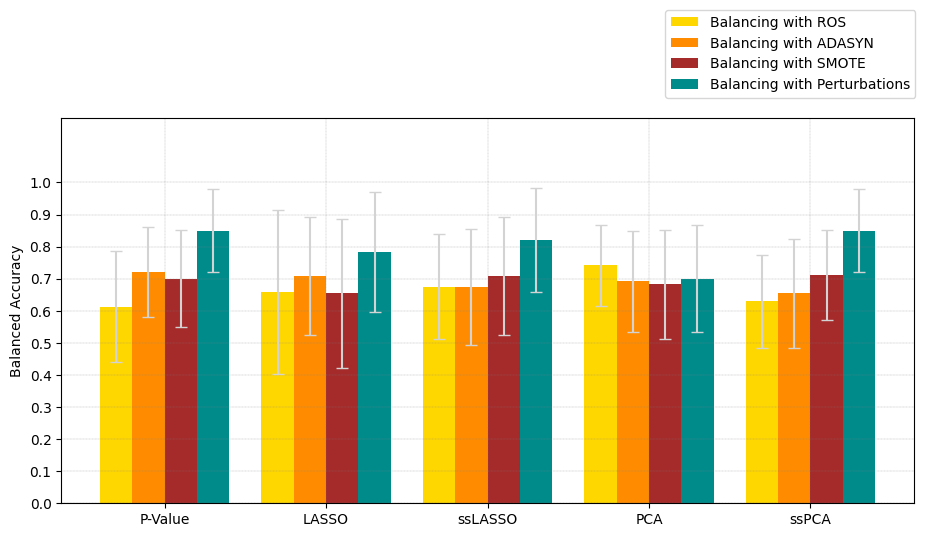

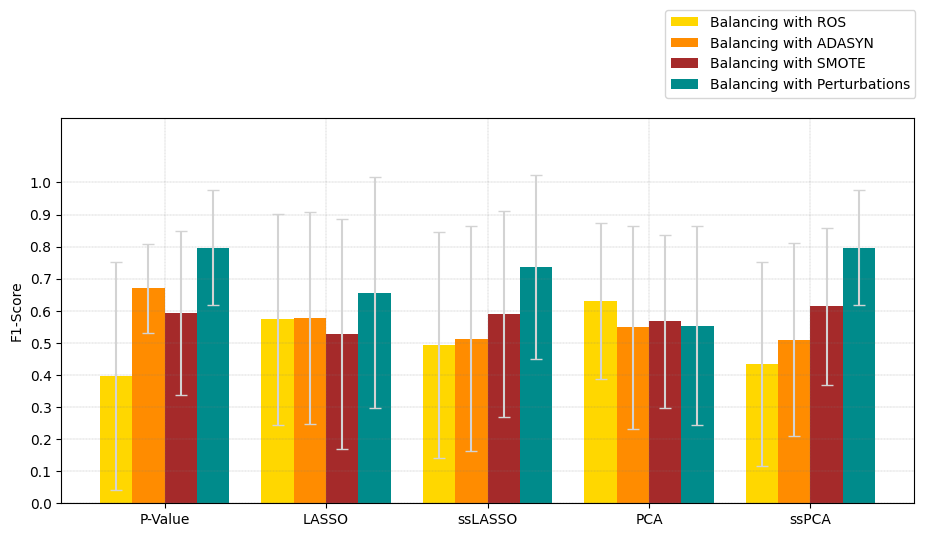


Fig.2: Mean (a) sensitivity, (b) specificity and (c) f1 score, averaged on the 10 test folds for different feature selection methods and a correlation threshold of 0.90, when differentiating CA from HCM, without augmentation (orange bars), using ROS (light yellow bars), ADASYN (light green bars) and SMOTE (dark green bars) or ROI perturbations (light blue bars). LASSO, least absolute shrinkage and selection operator; ssLASSO, semi-supervised LASSO; PCA, principal component analysis; ssPCA, semi-supervised PCA

(a) (b)

**
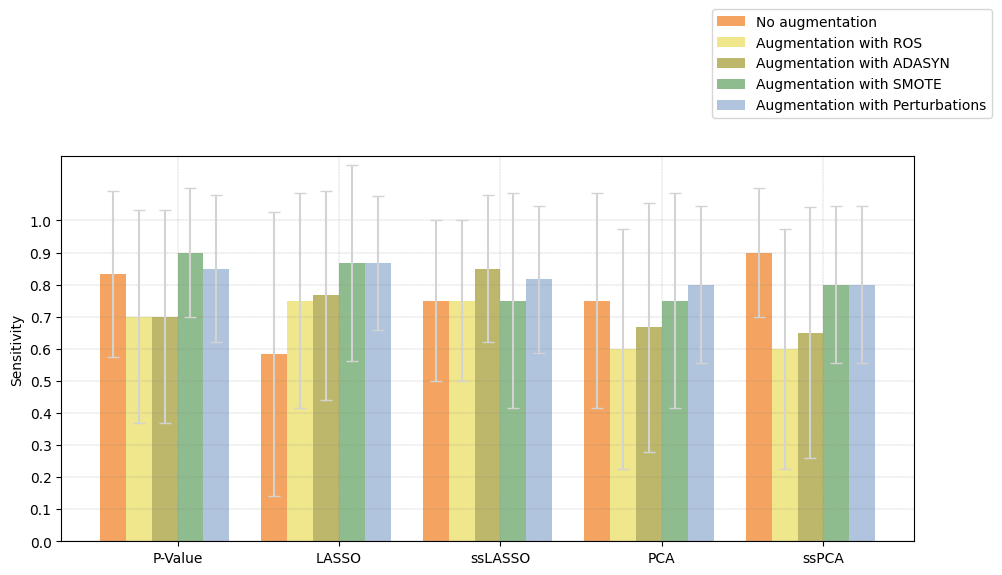

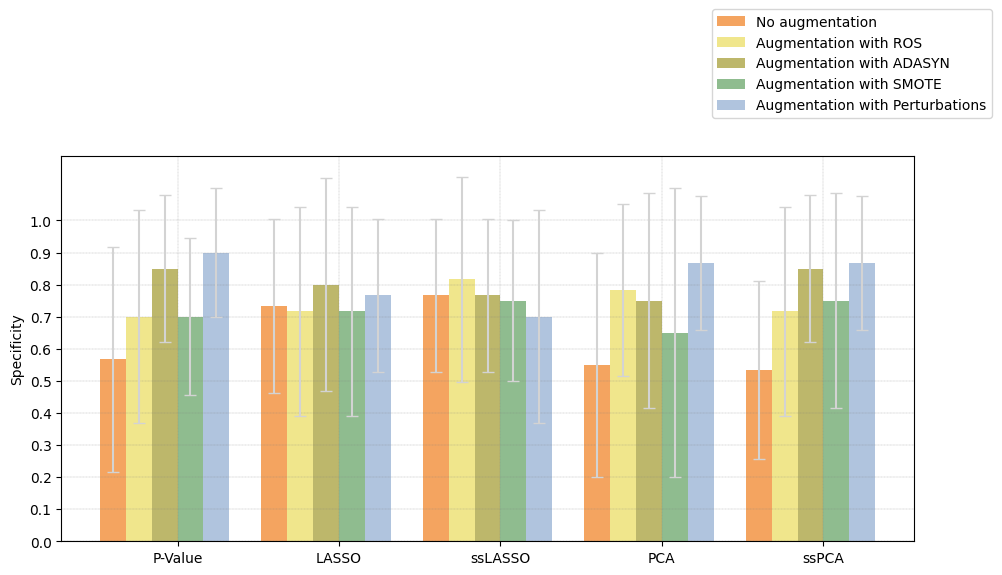
**

(c) (d)


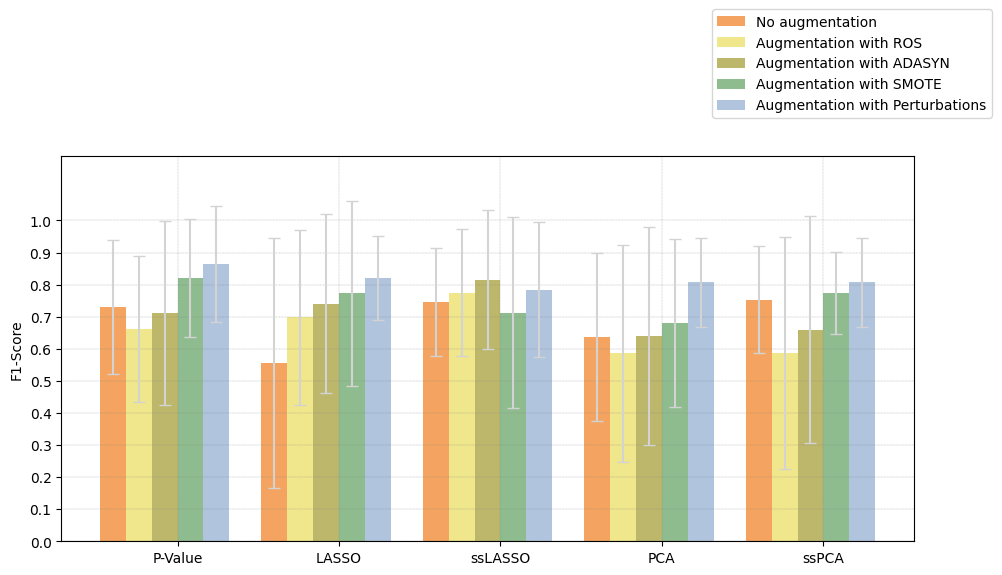

Supplement: Supplementary file 1 — Supplementary file1 (DOCX 410 KB) [file 10278_2024_1013_MOESM1_ESM.docx]
